# Predicting readmission and death after hospital discharge: a comparison of conventional frailty measurement with an electronic health record-based score

**DOI:** 10.1093/ageing/afab043

**Published:** 2021-03-25

**Authors:** Yong Yong Tew, Juen Hao Chan, Polly Keeling, Susan D Shenkin, Alasdair MacLullich, Nicholas L Mills, Martin A Denvir, Atul Anand

**Affiliations:** BHF Centre for Cardiovascular Science, University of Edinburgh, Edinburgh, UK; University of Edinburgh Medical School, Edinburgh, UK; University of Edinburgh Medical School, Edinburgh, UK; Geriatric Medicine Research Group, University of Edinburgh, Edinburgh, UK; Usher Institute, University of Edinburgh, Edinburgh, UK; Geriatric Medicine Research Group, University of Edinburgh, Edinburgh, UK; Usher Institute, University of Edinburgh, Edinburgh, UK; BHF Centre for Cardiovascular Science, University of Edinburgh, Edinburgh, UK; Usher Institute, University of Edinburgh, Edinburgh, UK; BHF Centre for Cardiovascular Science, University of Edinburgh, Edinburgh, UK; BHF Centre for Cardiovascular Science, University of Edinburgh, Edinburgh, UK; Geriatric Medicine Research Group, University of Edinburgh, Edinburgh, UK

**Keywords:** co-morbidity, frailty, older people, risk prediction

## Abstract

**Background:**

frailty measurement may identify patients at risk of decline after hospital discharge, but many measures require specialist review and/or additional testing.

**Objective:**

to compare validated frailty tools with routine electronic health record (EHR) data at hospital discharge, for associations with readmission or death.

**Design:**

observational cohort study.

**Setting:**

hospital ward.

**Subjects:**

consented cardiology inpatients ≥70 years old within 24 hours of discharge.

**Methods:**

patients underwent Fried, Short Physical Performance Battery (SPPB), PRISMA-7 and Clinical Frailty Scale (CFS) assessments. An EHR risk score was derived from the proportion of 31 possible frailty markers present. Electronic follow-up was completed for a primary outcome of 90-day readmission or death. Secondary outcomes were mortality and days alive at home (‘home time’) at 12 months.

**Results:**

in total, 186 patients were included (79 ± 6 years old, 64% males). The primary outcome occurred in 55 (30%) patients. Fried (hazard ratio [HR] 1.47 per standard deviation [SD] increase, 95% confidence interval [CI] 1.18–1.81, *P* < 0.001), CFS (HR 1.24 per SD increase, 95% CI 1.01–1.51, *P* = 0.04) and EHR risk scores (HR 1.35 per SD increase, 95% CI 1.02–1.78, *P* = 0.04) were independently associated with the primary outcome after adjustment for age, sex and co-morbidity, but the SPPB and PRISMA-7 were not. The EHR risk score was independently associated with mortality and home time at 12 months.

**Conclusions:**

frailty measurement at hospital discharge identifies patients at risk of poorer outcomes. An EHR-based risk score appeared equivalent to validated frailty tools and may be automated to screen patients at scale, but this requires further validation.

## Key Points

- Frailty measurement at hospital discharge identifies patients at risk of poorer outcomes over the next 12 months.
- An electronic health record (EHR) risk score performed similarly to validated frailty tools for predicting readmission or death.
- This EHR score is based on routine data and so could automate screening to identify patients at highest risk after discharge.
- This approach could be helpful for frailer inpatients managed outside of geriatric medicine, but this requires validation.

## Introduction

Despite increasing demands from our ageing population, allocated health resources for frail older patients are finite. As the balance of care shifts into community settings, targeting proactive geriatric assessment towards individuals at risk of a health or social crisis is of paramount importance. One approach to this challenge is to consider hospital admissions as an opportunity for intervention. In the hospitalised older population, functional decline despite resolution of acute illness is well recognised [1], but Comprehensive Geriatric Assessment (CGA) can improve the likelihood of independence after discharge. [2] However, non-specialists are increasingly managing frail patients in environments where access to a multidisciplinary team and effectiveness of CGA may be limited [3, 4].

Community-based interventions can improve function and reduce hospital readmissions [5], but these programmes are unrealistic to deliver unless highly targeted towards older adults with the greatest need. Objectively quantifying risk at the point of discharge may offer coordination between management of acute illness in hospital and prioritisation of functional recovery in the community. It may also help to recognise patients approaching the end of life, where proactive advanced care planning may be as important.

The concept of frailty is well recognised as a measure of vulnerability to dependency or death in the face of an acute stressor event [6, 7]. However, tools to measure frailty are numerous [8], often poorly correlate with each other [9, 10] and frequently require specialist equipment or specific bedside assessment. Despite guidelines advocating measurement of frailty in all older hospitalised patients to target CGA [11], this is rarely achieved. A recent survey of 121 NHS England hospital trusts reported that only 26% used standardised methodology to identify frailty. Better use of routinely collected data may help to overcome the clear barrier of additional measurement.

Many markers of frailty are recorded routinely within electronic health record (EHR) data. This is the basis of the electronic Frailty Index (eFI) that is now embedded into primary health records across the United Kingdom [12]. However, the eFI is infrequently available in secondary healthcare systems and will not rapidly update to reflect changes incurred by hospitalisation, so limiting its potential to guide early post-hospital care. Modern EHR systems include integrated alerting systems within the live clinical environment [13], providing a basis for routinely collected data to drive proactive care of older adults.

We studied frailty measurement at the point of hospital discharge from non-specialist geriatric care, to help identify patients at risk of unplanned readmission or death. We hypothesised that routine EHR data may provide a useful alternative to additional bedside frailty assessment.

## Methods

### Study design and participants

This study was performed on a cardiology inpatient ward at the Royal Infirmary of Edinburgh, United Kingdom. This unit was chosen as patients are not managed by specialist geriatric medicine services, either directly or via regular liaison input. Any patient under the clinical care of a cardiologist and aged ≥70 years old was eligible for inclusion. Convenience sampling was undertaken on days when researchers were available to review patients identified as ready for hospital discharge within the following 24 hours. Patients were excluded if any ongoing acute medical issues requiring inpatient management were subsequently identified or if unwilling or unable to provide informed consent. The study was conducted in accordance with the Declaration of Helsinki and approved by the Local Research Ethics Committee (reference 16/SS/0208). Written informed consent was obtained from all participants.

### Frailty measures

Four frailty measures were combined into a coordinated assessment taking ~15 minutes including completion of a brief questionnaire. The Fried phenotype was determined by standard methodology [14], combining measures of grip strength, gait speed, weight loss, exhaustion and low physical activity (1 point per trait, scale 0–5). The Short Physical Performance Battery (SPPB) combines gait speed measurement with chair rises and incremental standing balance tests (scale 0–12) [15]. Clinical nursing staff caring for participants completed the Clinical Frailty Scale (CFS), which uses descriptors to guide selection between ‘very fit’ to ‘terminally ill’ (scale 1–9) [16]. The PRISMA-7 is a self-rated 7-point questionnaire covering social support, mobility issues and activity limitation (scale 0–7) [17]. Detailed descriptions of each frailty tool are included in Supplementary Material A1.

### EHR risk score

Items for this measure were manually extracted from admission entry forms, laboratory results and healthcare utilisation information held within the hospital EHR (TrakCare; InterSystems Corporation, Cambridge, MA). This included 31 possible deficits (Supplementary Material A2) covering areas such as co-morbidity, mobility, continence, falls, low body weight, polypharmacy (>4 prescribed medications), anaemia, hypoalbuminaemia and requirement for carer support. These variables were chosen as plausible markers of frailty that are available as part of routine and nationally reportable healthcare activity. Priority was given to completeness of data available in pilot testing and measures undertaken as part of national Excellence in Care reporting standards [18] that are likely to be generalisable to other healthcare systems. In keeping with frailty indices [19], the EHR risk score was expressed as a proportion of available deficits (e.g. if eight deficits present—8/31 = 0.26).

### Co-morbidity

A count of co-morbid conditions was obtained from electronic and paper health records. Included conditions were ischaemic heart disease, atrial fibrillation, diabetes mellitus, stroke, cancer, heart failure, peripheral vascular disease, asthma, chronic obstructive pulmonary disease, chronic kidney disease and dementia. As all measures were based on discharge status, newly acquired co-morbid conditions from the study hospitalisation episode were included.

### Outcomes

A linked regional EHR was used to determine the primary endpoint of readmission or all-cause mortality at 90 days following assessment. Secondary outcomes were all-cause mortality and ‘home time’ in the 12 months following discharge. Home time describes the number of days spent alive and out of hospital, so accounting for the burden of multiple or prolonged hospital readmissions and early death. It is calculated by subtracting the number of days to death and/or the total number of days in all unscheduled hospital readmissions from 365 days. All patients in this study completed electronic follow-up to 1 year.

### Statistical analysis

Continuous data are presented as means ± standard deviation (SD) or median ± interquartile range and where appropriate compared by Student’s *t*-test, Mann–Whitney *U* test or analysis of variance. Categorical data are presented as absolute numbers (percentages) and compared by Chi-squared test. Agreement between frailty tools was compared by Cohen’s Kappa testing using previously described thresholds to define frailty. Cox proportional hazards regression modelling was undertaken for readmission and death. As the scale of each tool varied, modelling outputs by unit change would not be comparable. For this reason, continuous variables were first standardised, including age, co-morbidity count, all frailty measures and the EHR risk score. Where co-morbidity was added to models as a covariate, the total count of conditions was included rather than individual disease states. The SPPB was reversed to allow easier comparison, as this tool associates higher scores with better function in contrast to all other measures tested.

For the home time analysis, co-morbidity, frailty tools and the EHR risk score were divided into low-, medium- and high-risk groups attempting to create units of approximately equal size or using defined cut-points where previously validated for a frailty tool (Supplementary Material A3). Comparisons of home time days between groups were undertaken using Wilcoxon pairwise-comparison tests with correction for multiple testing. Linear regression modelling was used to describe the adjusted change in home time by risk group, using the lowest risk group as the reference. All analyses were completed with R (version 3.6.0).

## Results

A total of 186 patients participated between January 2017 and April 2018 (64% males, mean age 79 ± 6 years). Baseline characteristics are shown in Table 1. The primary outcome of 90-day readmission or death occurred in 55 (30%) patients, of whom 7 (4%) had died. Patients with the primary outcome were more co-morbid (mean 2.6 ± 1.6 vs. 2.1 ± 1.5 chronic conditions, *P* = 0.03) and had higher total medication use (10 ± 4 vs. 8 ± 4 medications, *P* = 0.01) than patients surviving without readmission. Whilst age, sex and body mass index (BMI) were similar between groups, patients with the primary outcome scored higher by Fried (2.3 ± 1.4 vs. 1.8 ± 1.2, *P* = 0.005), CFS (3.7 ± 1.4 vs. 3.2 ± 1.3, *P* = 0.04) and EHR risk scores (0.26 ± 0.10 vs. 0.21 ± 0.10, *P* = 0.004) compared with those who survived without readmission. The SPPB and PRISMA-7 frailty measures did not differ between these groups. At previously reported threshold scores to identify frailty, agreement between the four validated tools showed only fair agreement or lower by Cohen’s Kappa (Supplementary Material A4).

**Table 1:** Baseline characteristics

	All	90-day composite			12 months		
		Yes	No	*P*-value	Dead	Alive	*P*-value
Number	186	55	131		21	165	
Males	119 (64)	33 (60)	86 (66)	0.57	13 (62)	106 (64)	1
Age, years	79 (6)	80 (7)	79 (6)	0.21	82 (6)	79 (6)	0.06
BMI, kg/m^2^	27.5 (6.2)	26.2 (6.3)	28.1 (6.1)	0.07	25.4 (5.1)	27.8 (6.3)	0.13
**Co-morbidities**							
IHD	79 (43)	25 (46)	54 (41)	0.71	7 (33)	72 (44)	0.51
Atrial fibrillation	79 (42)	30 (55)	49 (38)	0.05	16 (76)	63 (38)	0.002
Diabetes mellitus	52 (28)	14 (26)	38 (29)	0.75	4 (19)	48 (29)	0.48
Stroke	30 (16)	10 (18)	20 (15)	0.78	5 (24)	25 (15)	0.48
Cancer	33 (18)	12 (22)	21 (16)	0.46	5 (24)	28 (17)	0.64
Heart failure	52 (28)	17 (31)	35 (27)	0.69	7 (33)	45 (27)	0.75
PVD	18 (10)	5 (9)	13 (10)	1	1 (5)	17 (10)	0.68
Asthma/COPD	37 (20)	13 (24)	24 (18)	0.53	6 (29)	31 (19)	0.44
CKD	27 (15)	13 (24)	14 (11)	0.04	4 (19)	23 (14)	0.77
Dementia	4 (2)	3 (6)	1 (1)	0.15	1 (5)	3 (2)	0.94
Co-morbidity count	2.2 (1.6)	2.6 (1.6)	2.1 (1.5)	0.03	2.7 (1.5)	2.2 (1.6)	0.15
Total medications	8 (4)	10 (4)	8 (4)	0.01	7 (3)	9 (5)	0.19
**Blood results**							
Haemoglobin, g/l	130 (19)	126 (20)	132 (18)	0.1	131 (25)	130 (18)	0.87
Creatinine, mmol/l	98 (40)	107 (53)	94 (31)	0.04	122 (72)	96 (34)	0.007
Albumin, g/l	35 (5)	34 (4)	35 (5)	0.31	35 (5)	35 (5)	0.89
**Frailty measures**							
Fried	1.9 (1.3)	2.3 (1.4)	1.8 (1.2)	0.005	2.6 (1.3)	1.8 (1.2)	0.006
CFS	3.3 (1.4)	3.7 (1.4)	3.2 (1.3)	0.04	4.2 (1.6)	3.2 (1.3)	0.002
SPPB	5.5 (3.7)	5.1 (3.5)	5.7 (3.8)	0.38	4.1 (3.4)	5.7 (3.7)	0.06
PRISMA-7	3.0 (1.3)	3.2 (1.4)	2.9 (1.2)	0.25	3.4 (1.1)	3.0 (1.3)	0.17
EHR risk score	0.22 (0.11)	0.26 (0.10)	0.21 (0.10)	0.004	0.27 (0.11)	0.22 (0.10)	0.02

All measures are mean (SD) or number (%).

The 90-day composite primary outcome included readmission or death.

IHD, ischaemic heart disease; PVD, peripheral vascular disease; COPD, chronic obstructive pulmonary disease; CKD, chronic kidney disease.

Fried, CFS and EHR risk scores were independently associated with the primary outcome after addition to a base model including age, sex and co-morbidity (Table 2). Using standardised adjusted hazard ratios (aHR), the effect size per SD increase was greatest for Fried (aHR 1.47, 95% confidence interval [CI] 1.18–1.81, *P* < 0.001). The CFS (aHR 1.24, 95% CI 1.01–1.51, *P* = 0.04) and EHR risk scores (aHR 1.35, 95% CI 1.02–1.78, *P* = 0.04) had similar performance. In all models, age and sex were not associated with readmission or death once frailty measures or the EHR risk score were included.

**Table 2:** Cox regression models for 90-day readmission or death

Variable	Model 1	Model 2	Model 2 +				
			Fried	CFS	SPPB	PRISMA-7	EHR score
Age	1.23^*^ **(1.02–1.48)**	1.22^*^ **(1.01–1.48)**	1.14 (0.95–1.39)	1.14 (0.93–1.39)	1.19 (0.98–1.44)	1.16 (0.95– 1.41)	1.16 (0.95–1.40)
Male sex	0.92 (0.63–1.35)	0.86 (0.59–1.27)	1.06 (0.70–1.58)	0.91 (0.62–1.34)	0.94 (0.62–1.43)	0.83 (0.56– 1.23)	0.94 (0.64–1.40)
Co-morbidity		1.32^*^^*^ **(1.11–1.56)**	1.21^*^ **(1.01–1.44)**	1.26^*^^*^ **(1.06–1.50)**	1.28^*^^*^ **(1.07–1.52)**	1.28^*^^*^ **(1.08–** **1.53)**	1.06 (0.82–1.38)
							
Frailty tool			1.47^*^^*^^*^ **(1.18–1.81)**	1.24^*^ **(1.01–1.51)**	1.13 (0.91–1.40)	1.17 (0.95– 1.45)	1.35^*^ **(1.02–1.78)**

Age, co-morbidity and frailty measures have been standardised, with the output representing the HR (95% CI) for 90-day readmission or death per SD increase in each variable. For clearer comparison with other measures, the SPPB score has been reversed, such that increasing values represent greater impairment.

Significance of each model output denoted by ^*^P < 0.05, ^*^^*^P < 0.01, ^*^^*^^*^P < 0.001. Results in bold are significant to at least p < 0.05

By 12 months of follow-up, 21 (11.3%) patients had died. These individuals were more likely to have atrial fibrillation (76% of those who died vs. 38% of survivors, *P* = 0.002), but age and the total count of co-morbid conditions otherwise did not differ (Table 1). Fried scores (2.6 ± 1.3 vs. 1.8 ± 1.2, *P* = 0.006), CFS (4.2 ± 1.6 vs. 3.2 ± 1.3, *P* = 0.002) and EHR risk scores (0.27 ± 0.11 vs. 0.22 ± 0.10, *P* = 0.02) were higher in those who died than in survivors. The SPPB and PRISMA-7 did not differ between these groups. In modelling for death at 12 months, all tested measures were independently associated with mortality after adjustment for age, sex and co-morbidity (Table 3). In each of these models, co-morbidity was not independently associated with death once a frailty measure or the EHR risk score had been included.

**Table 3:** Cox regression models for 12-month mortality

Variable	Model 1	Model 2	Model 2 +				
			Fried	CFS	SPPB	PRISMA-7	EHR score
Age	1.55^*^ **(1.06–2.26)**	1.53^*^ **(1.05–2.24)**	1.45 (0.98–2.14)	1.36 (0.91–2.02)	1.42 (0.96–2.09)	1.34 (0.91– 1.96)	1.38 (0.93–2.03)
Male sex	1.03 (0.48–2.24)	1.00 (0.46–2.18)	1.27 (0.57–2.80)	1.07 (0.49–2.34)	1.43 (0.63–3.29)	0.87 (0.39– 1.93)	1.09 (0.50–2.39)
Co-morbidity		1.20 (0.85–1.69)	1.07 (0.73–1.55)	1.13 (0.79–1.63)	1.11 (0.78–1.60)	1.12 (0.77– 1.61)	0.79 (0.47–1.33)
							
Frailty tool			1.85^*^^*^ **(1.26–2.71)**	1.56^*^ **(1.07–2.27)**	1.79^*^ **(1.14–2.82)**	1.50^*^ **(1.00–** **2.25)**	1.81^*^ **(1.04–3.13)**

Age, co-morbidity and frailty measures have been standardised, with the output representing the HR (95% CI) for 12-month mortality per SD increase in each variable. For clearer comparison with other measures, the SPPB score has been reversed, such that increasing values represent greater impairment.

Significance of each model output denoted by ^*^P < 0.05, ^*^^*^P < 0.01, ^*^^*^^*^P < 0.001. Results in bold are significant to at least p < 0.05

**Figure 1:**
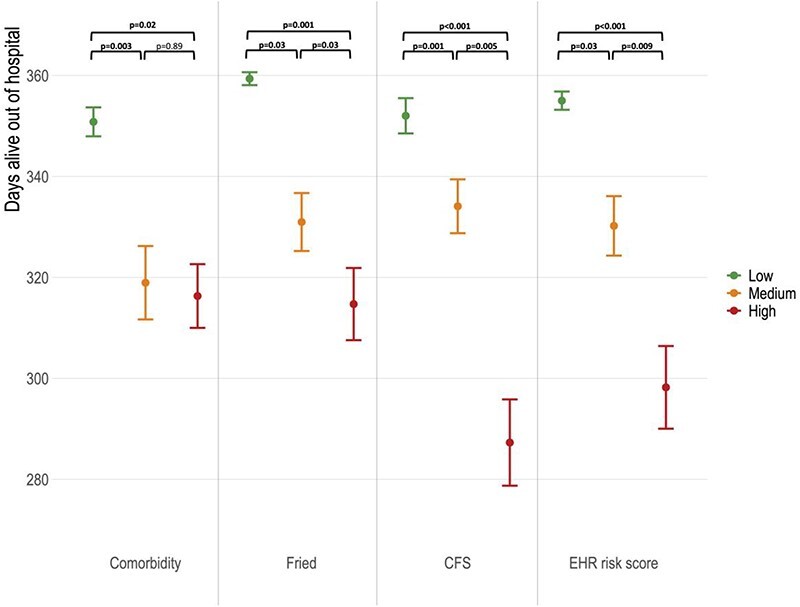
Difference in home time in the year following hospital discharge by low, medium and high-risk co-morbidity and frailty tool classifications. Figures presented are mean number of days alive out of hospital at 12 months ± standard error of mean. Between group significance testing undertaken by Wilcoxon pairwise-comparison with correction for multiple testing. Classification of risk groups and detailed modelling outputs are presented in Supplementary Material A3.

Over the 12 months following frailty assessment, 84 (45%) patients had experienced at least one unscheduled hospital readmission. The mean home time across the whole cohort was 330 days. The relationship between home time and low-, medium- and high-risk groups for co-morbidity, Fried, CFS and EHR risk scores is shown in Figure 1. Patients in the lowest risk co-morbidity group (0 or 1 chronic condition) experienced ~30 additional days alive and out of hospital over the year of follow-up, compared with those with two or more chronic conditions. However, no further reductions in home time were observed with higher levels of multimorbidity. In contrast, the Fried, CFS and EHR risk score all showed separation in home time between low-, medium- and high-risk groups (group differences all *P* < 0.05 after multiple testing correction).

In linear regression models, patients in the highest risk groups for each frailty tool and the EHR risk score experienced falls in home time compared with low-risk patients. The associations persisted after adjustment for age and sex using the EHR risk score (50-day reduction, 95% CI –8 to −92 days, *P* = 0.02), CFS (58-day reduction, 95% CI –22 to −93 days, *P* = 0.002) and SPPB (35-day reduction, 95% CI –2 to −67 days, *P* = 0.04). The cut-offs for each tool and full modelling outputs are available in Supplementary Material A3.

## Discussion

This study has compared conventional frailty measurement with an EHR-based score at hospital discharge in relation to outcomes for older adults. There are several key observations. First, a count of deficits from routinely collected EHR data performed similarly to the Fried frailty phenotype and CFS in its association with early readmission or death, independent of age, sex and co-morbidity. Second, more intensive physical measures (SPPB) and self-reported frailty (PRISMA-7) were not informative for this outcome. Third, all frailty measures and the EHR risk score were associated with 12-month mortality, whereas co-morbidity appeared less important in this estimation. Finally, patients with higher CFS, SPPB and EHR risk scores experienced fewer days alive and out of hospital over the year following discharge.

The association between hospital frailty and readmission or death has been previously recognised [20, 21]. However, these measures often use tools requiring additional bedside assessment, bespoke data collection or equipment and most ascertain frailty at the point of hospital admission. Functional status is increasingly recognised as a ‘6th vital sign’ and acquired disability during a hospital admission is a clear risk marker for future poor health outcomes [1, 22]. There are plausible reasons why measuring frailty and function at the point of hospital discharge could be useful, to account for the heterogeneity of illness and functional recovery. This may provide an updated assessment of an individual’s reserve at the end of acute care. Ultimately a risk tool only has value if it helps target interventions that improve outcomes. Community-based trials offer hope that recovery after hospital admission may be modified, through targeted physical activity programmes such as those shown to delay new disability in the LIFE study [23, 24].

It is perhaps unsurprising that those with the greatest deficits do substantially worse in their post-hospital recovery. However, measurement of risk, communication and future care planning are not ingrained into routine healthcare outside of geriatric medicine. Only a quarter of older adults admitted to hospital in England are screened for frailty. [25] A recently published study by Blomaard *et al.* [26] assessed the feasibility of a screening risk assessment tool for older patients in the emergency department. Whilst completion rates were encouraging, the probability of screening fell by 37% when the department was busy and by 45% in the sickest patients. This suggests that manual screening approaches are prone to system pressures, which have perhaps become even more apparent during the COVID-19 pandemic. A further concern in older patients is the lack of validity of physical frailty instruments in patients with cognitive impairment or delirium, which complicates more than one in five medical admissions. [27] Using routinely collected EHR data to derive an automated discharge risk score has potential to remove measurement barriers without additional financial or time costs. Our study suggests that such an approach is feasible and retains similar performance to validated frailty measures.

There are existing EHR measures of frailty that predict hospital admission and death. Most notable in the UK are the eFI [12] from primary care and Hospital Frailty Risk Score from secondary care data. [28] Whilst both are helpful for population-based studies, these indices require coding of completed hospital episodes and so cannot be used for real-time risk prediction at discharge. Readmission risk scores have also been described using routinely available administrative data. However, a systematic review of 26 such models described overall performance as ‘poor’, noting that ‘few considered mental health, functional status and social determinant variables’ [29]. The recording of basic markers of function, frailty and cognition within live hospital systems is already widespread and will only grow further. Our EHR risk score suggests how these measures may be combined to better understand trajectories of recovery from acute illness and maximise the value of recent digital health transformations.

It is important that studies including older people report patient-centred endpoints. Numerous qualitative studies have identified the minimisation of dependence and disability as a priority for older people [30–32]. This is a challenge in many forms of research, particularly those that rely on EHR follow-up, where the integration of patient-reported outcome measures is limited, although progressively improving [33]. Patients with cognitive impairments and lower health literacy may be excluded without carefully considered methods. We have reported home time as an endpoint from routine data that reflects some of the burden of prolonged or repeated readmissions that frequently complicate healthcare for older adults. It is encouraging that the EHR risk score, alongside validated frailty measurement tools, identified patients at highest risk of fewer home time days.

We recognise the limitations inherent with a single-centre observational cohort study. Validation in an external cohort with a larger study population would be informative. We limited our study to cardiology patients as a model of non-specialist geriatric care, but testing in a wider range of specialities would be useful. Not all hospital EHRs will collect the same frailty markers, but similar measures frequently form part of quality assurance standards for hospital care. As a consented patient study, only those deemed to have capacity to consent were included. Although a small number of patients with early dementia were able to participate, this does not fully reflect the older hospital population, a factor that may also favour the use of EHR data to enable equitable risk assessment of all patients. We recognise that our study endpoints were focussed on mortality and healthcare utilisation, which should be broadened in future studies to reflect a wider range of harmful outcomes relevant to older people such as falls, functional and cognitive decline.

In summary, this preliminary study has shown the potential for routinely collected EHR data to inform risk assessment at the point of hospital discharge. This approach appeared equivalent to well-validated frailty measures that require additional time and equipment to obtain. Automated EHR-based scores now require testing at scale, using a broader range of holistic outcome measures to define threshold scores for risk that could be amenable to improved care pathways. Such a tool could efficiently target intervention trials to reduce harm to older patients after hospital discharge.

## Supplementary Material

aa-20-1268-File001_afab043
