# Supplementary material for: Predicting readmission and death after hospital discharge: a comparison of conventional frailty measurement with an electronic health record-based score
Source: Age Ageing. 2021 Mar 25;50(5):1641–8. doi: 10.1093/ageing/afab043 (PMC8437069; doi:10.1093/ageing/afab043)
Supplement: aa-20-1268-File001_afab043 [file aa-20-1268-file001_afab043.docx]

Predicting readmission and death after hospital discharge: a comparison of conventional frailty measurement with an electronic health record-based score

**SUPPLEMENTARY DATA**

- **Appendix 1:** Description of Frailty Tools
- **Appendix 2:** EHR risk score components
- **Appendix 3:** Home time modelling and definitions
- **Appendix 4:** Agreement between validated frailty tools

**Appendix 1: Description of Frailty Tools**

***Fried Phenotype***

The Fried phenotype was obtained using two physical measures and three questionnaire responses to ascertain five components.[1] Grip strength was assessed by three trials (two dominant hand, one non-dominant hand) of maximal unsupported hand grip strength assessed using a Jamar digital dynamometer. This machine provides a maximal strength measure obtained on each trial to the nearest 0.1kg. The associated phenotypic trait of weakness was allocated if participants achieved lower than the 20^th^ percentile derived from population data adjusted for gender and body mass index. If a participant was unable to complete the grip strength assessment they were allocated a mark for weakness.

Gait speed was assessed using an unobstructed 5 metre track marked by cones, with a clear 1 metre runoff at each end. Participants started in the runoff area and were asked to walk at their normal pace past the farthest away cone using their own walking aids if required. Timing was taken by stopwatch for the period the participant was travelling between the two cone markers. Three trials were undertaken with each participant, with the mean of these three recordings used to calculate a gait speed in metres per second. The associated phenotypic trait of slowness was allocated if participants achieved lower than the 20^th^ percentile gait speed derived from population data adjusted for gender and height. If a participant was unable to complete all three trials they were allocated the slowness marker.

Shrinking was assessed by a positive questionnaire response for self-reported weight loss:

*“Have you unintentionally lost more than 10lbs (4.5kg) in weight in the last year?”*

Exhaustion was assessed using responses to two statements from the Centre for Epidemiology Studies Depression (CES-D) scale[2]: *“I felt that everything I did was an effort in the last week”* and *“I could not get going in the last week”*. Participants were provided with four options for each statement and asked to pick the single response that best fitted their experience. A response of “moderate amount of the time (3-4 days per week)” or more frequent for either question resulted in allocation of an exhaustion trait.

For determining low physical activity, participants were asked if, in the last 3 months, they either (a) did not perform any weight bearing physical activity, (b) spent more than 4 hours continuously sitting per day, or (c) went for a short walk once per month or less. The presence of any of these markers were used to allocate the low physical activity trait to a participant. This is a modification of definition used by Fried *et al.* in the original description of the phenotype, but is consistent with use in large frailty studies such as the Invecchiare in Chianti study[3] and the Frailty Intervention Trial.[4] The original Fried phenotype used the Minnesota leisure time physical activity questionnaire.[5] This content was considered less relevant for non-American respondents and the length of the questionnaire was also prohibitive.

As described by Fried *et al.*, individuals with 3 or more traits were considered frail (high-risk), with 1-2 traits pre-frail (medium-risk) and 0 traits not frail (low-risk).

***Clinical Frailty Scale***

The CFS is a structured scale of descriptors to guide selection between nine levels ranging from “very fit: 1” to “terminally ill: 9”. It requires no physical measures or formal questionnaire responses, but knowledge of the participant is required. Assessment criteria include activity, symptoms and assistance usually required with personal activities of daily living (e.g. washing and toileting), and instrumental tasks necessary for independent community living (e.g. managing finances and medications). Frailty may be assessed as a continuum, but is considered present at a score ≥5 (high-risk), with vulnerability or pre-frailty at 4 points.[6] Clinic nursing staff completed the CFS based on their professional assessment, discussion with the participant or family and any documentation of premorbid functional status.

***PRISMA-7***

This is a questionnaire-based measure of frailty is actually a case-finding tool for disability. It is simple to complete with just 7 questions, two of which do not require the patient (age and sex). The questions focus on self-perceived limitations and health problems as well as questions related to requirement for help walking and with activities of daily living. All questions are answered ‘yes’ or ‘no’ and the total PRISMA-7 score is the sum of positive responses. The authors described a cut-off for high-risk at ≥3 points.[7]

***Short Physical Performance Battery***

The SPPB comprises three tests of lower extremity function: gait speed, standing balance and chair rises. Each element scores a maximum of 4 points, with a lower score indicating greater impairment and 0 points an inability to complete the task. The gait speed scoring allocation in the original description of the SPPB was based on the time taken to walk an 8 foot track.[8, 9] To reduce repetitive testing, these cut-off times were converted into a speed in metres per second and points were allocated using the mean measure of gait speed obtained from the Fried phenotype testing across three trials of a 5 metre walk.

For standing balance, participants were sequentially asked to maintain their feet in side-by-side, semi-tandem and tandem positions for 10 seconds each without the use of external balancing aids. Assessment was stopped at the point at which a participant was unable to complete a full 10 second balance. Participant safety was a priority and testing was performed with a member of the research team in close proximity on either side of the participant for support in case of imbalance.

For the final component of the SPPB, participants were asked to rise from a chair with their arms folded across their chest, so as not to use their upper limbs or chair arms for propulsion. If a participant was able to complete this task, they were timed standing up and sitting down in this manner 5 times as quickly as possible. Scores between 1–4 were allocated according to cut-offs defined by the study authors[9], with 0 points for those unable to complete all 5 chair rises.

The sum total score out of 12 points reflects higher scores with better function, unlike all the other frailty tools being examined. To allow easier comparison in modelling outputs, the direction of the total SPPB score was reversed.

**Appendix 2: EHR risk score components**

The following 31 components were included as ‘deficits’ in the EHR risk score. The table shows the number of cases and proportion with each deficit in the study sample (total sample size 186 patients). Items were extracted from multiple forms (structured questionnaires), laboratory results and measurements held within the local EHR. Where items were recorded at multiple timepoints, the latest measure (closest to discharge) was used. All deficits are equally weighted in the final EHR risk score.

| **Frailty Marker** | **n** | **%** |
| --- | --- | --- |
| Lives alone | 79 | 42 |
| Impaired vision | 4 | 2 |
| Impaired hearing | 18 | 10 |
| Difficulty sleeping | 9 | 5 |
| Leg ulceration | 5 | 3 |
| Requiring assistance with transfers | 12 | 6 |
| Requiring assistance with walking (aid or person) | 66 | 35 |
| Incontinence | 5 | 3 |
| Requiring package of care | 25 | 13 |
| Requiring assistance with personal care | 20 | 11 |
| BMI <18kg/m^2^ | 5 | 3 |
| Serum albumin <35g/L | 68 | 37 |
| Haemoglobin <120g/L (female) or <130g/L (male) | 75 | 40 |
| Estimated glomerular filtration rate (eGFR) <60ml/min/1.73m^2^ | 57 | 31 |
| Total number of prescribed medications ≥5 at discharge | 162 | 87 |
| Hospital admission >7 days | 67 | 36 |
| ≥2 hospital admissions in the last 12 months | 44 | 24 |
| History of falls | 21 | 11 |
| Hypertension | 117 | 63 |
| Myocardial infarction | 79 | 42 |
| Arrhythmia including atrial fibrillation | 79 | 42 |
| Diabetes | 52 | 28 |
| Stroke | 30 | 16 |
| Any history of cancer | 33 | 18 |
| Heart failure | 52 | 28 |
| Peripheral vascular disease | 18 | 10 |
| Obstructive airways disease (COPD or asthma) | 37 | 20 |
| Arthritis (osteoarthritis or rheumatoid) | 24 | 13 |
| Alcohol excess | 8 | 4 |
| Psychiatric diagnosis (not dementia) | 19 | 10 |
| Dementia | 4 | 2 |

**Appendix 3: Home time modelling and definitions**

The table below summarises the definition of ‘low’, ‘medium’ and ‘high’ risk groups for comorbidity, each frailty tool and the EHR risk score. Linear regression modelling shows the unadjusted change in days alive out of hospital (home time) in medium and high-risk groups when compared to the referent (low risk) patients. The adjusted model includes age and sex.

| **Tool** | **Risk** | **Definition** |  | **Change in Home Time** | |
| --- | --- | --- | --- | --- | --- |
|  |  |  | **n** | **Unadjusted** | **Adjusted** |
| Comorbidity | Low | 0-1 conditions | 68 | Ref | Ref |
|  | Medium | 2-3 conditions | 79 | **–32 (–6 to –58)*** | **–33 (–7 to –59)*** |
|  | High | ≥4 conditions | 39 | **–35 (–3 to –66)*** | **–36 (–5 to –57)*** |
|  |  |  |  |  |  |
| Fried | Low | Score 0 | 26 | Ref | Ref |
|  | Medium | Score 1-2 | 104 | –28 (+6 to –63) | –22 (+13 to –57) |
|  | High | Score ≥3 | 56 | **–45 (–7 to –82)*** | –37 (+1 to -76) |
|  |  |  |  |  |  |
| CFS | Low | Score 1-2 | 52 | Ref | Ref |
|  | Medium | Score 3-4 | 98 | –18 (+8 to –44) | –14 (+13 to –41) |
|  | High | Score ≥5 | 36 | **–65 (–32 to –98)***** | **–58 (–22 to –93)**** |
|  |  |  |  |  |  |
| EHR Score | Low | 0 – 0.12 | 31 | Ref | Ref |
|  | Medium | 0.12 – 0.35 | 130 | –25 (+6 to –56) | –20 (+12 to –51) |
|  | High | ≥0.36 | 25 | **–57 (–15 to –99)**** | **–50 (–8 to –92)*** |
|  |  |  |  |  |  |
| PRISMA-7 | Low | Score 0-1 | 20 | Ref | Ref |
|  | Medium | Score 2 | 55 | –26 (+15 to –66) | –34 (+11 to –79) |
|  | High | Score ≥3 | 111 | **–39 (–1 to –77)*** | –41 (+3 to –85) |
|  |  |  |  |  |  |
| SPPB | Low | Score ≥9 | 42 | Ref | Ref |
|  | Medium | Score 4-8 | 77 | –20 (+10 to –50) | –17 (+14 to –47) |
|  | High | Score 0-3 | 67 | **–41 (–11 to –72)**** | **–35 (–2 to –67)*** |

The change in home time measure presented is the beta value from unadjusted and adjusted (age/sex) linear regression models including the listed tool as an explanatory variable. This represents the change in days (95% CI) for medium and high-risk patients compared to the reference group of low-risk individuals.

Significance of each model output denoted by * <0.05, ** <0.01, *** <0.001. Emboldened results are significant.

Abbreviations: CFS = Clinical Frailty Scale; EHR = Electronic Health Record; SPPB = Short Physical Performance Battery

**Appendix 4: Agreement between validated frailty tools**

|  | **Fried** | **CFS** | **SPPB** | **PRISMA-7** |
| --- | --- | --- | --- | --- |
| **Fried** |  |  |  |  |
| **CFS** | 0.26  (0.09–0.43) |  |  |  |
| **SPPB** | 0.32  (0.19–0.46) | 0.32  (0.18–0.46) |  |  |
| **PRISMA-7** | 0.23  (0.10–0.36) | 0.14  (0.02–0.27) | 0.26  (0.12–0.40) |  |

Cohen’s Kappa statistic for agreement between each pair of tests shown. Values are central estimate (95% confidence interval). Abbreviations: CFS = Clinical Frailty Scale; SPPB = Short Physical Performance Battery. Thresholds used to define frail or high-risk are Fried ≥3 points, CFS ≥5 points, SPPB ≤5 points, PRISMA-7 ≥3 points.

**References**

1. Fried LP, Tangen CM, Walston J, et al. Frailty in older adults: Evidence for a phenotype. *The Journals of Gerontology Series A: Biological Sciences and Medical Sciences*. 2001;56(3):M146-M156.

2. Orme JG, Reis J, Herz EJ. Factorial and discriminant validity of the Center for Epidemiological Studies Depression (CES-D) scale. *J Clin Psychol*. 1986;42(1):28-33.

3. Cesari M, Leeuwenburgh C, Lauretani F, et al. Frailty syndrome and skeletal muscle: results from the Invecchiare in Chianti study. *Am J Clin Nutr*. 2006;83(5):1142-1148.

4. Fairhall N, Aggar C, Kurrle SE, et al. Frailty Intervention Trial (FIT). *BMC Geriatrics*. 2008;8(1):27. doi:10.1186/1471-2318-8-27.

5. Taylor HL, Jacobs DR, Schucker B, Knudsen J, Leon AS, Debacker G. A questionnaire for the assessment of leisure time physical activities. *J Chronic Dis*. 1978;31(12):741-755.

6. Rockwood K, Song X, MacKnight C, et al. A global clinical measure of fitness and frailty in elderly people. *CMAJ*. 2005;173(5):489-495. doi:10.1503/cmaj.050051.

7. Raiche M, Hebert R, Dubois MF. PRISMA-7: a case-finding tool to identify older adults with moderate to severe disabilities. Arch Gerontol Geriatr. 2008 Jul-Aug;47(1):9-18.

8. Guralnik JM, Simonsick EM, Ferrucci L, et al. A short physical performance battery assessing lower extremity function: association with self-reported disability and prediction of mortality and nursing home admission. *J Gerontol*. 1994;49(2):M85-M94.

9. Guralnik JM, Ferrucci L, Simonsick EM, Salive ME, Wallace RB. Lower-extremity function in persons over the age of 70 years as a predictor of subsequent disability. *N Engl J Med*. 1995;332(9):556-561.
